# The interaction of genetics and physical activity in the pathogenesis of metabolic dysfunction associated liver disease

**DOI:** 10.1038/s41598-024-68271-4

**Published:** 2024-08-01

**Authors:** Hanna Frostdahl, Nouman Ahmad, Ulf Hammar, Andrés Martínez Mora, Taro Langner, Tove Fall, Joel Kullberg, Håkan Ahlström, Hannah L. Brooke, Shafqat Ahmad

**Affiliations:** 1 Molecular Epidemiology, Department of Medical Sciences, Uppsala University, Uppsala, Sweden; 2 Radiology, Department of Surgical Sciences, Uppsala University, Uppsala, Sweden; 3 Antaros Medical AB, BioVenture Hub, Mölndal, Sweden; 4 Medical Epidemiology, Department of Surgical Sciences, Uppsala University, Uppsala, Sweden; 5 Preventive Medicine Division, Brigham and Women’s Hospital, Harvard Medical School, Boston, MA USA

**Keywords:** MASLD, CLD, Liver fat, Liver volume, IPAQ, Gene-environment interactions, Liver cancer, Non-alcoholic fatty liver disease

## Abstract

Genetic variants associated with increased liver fat and volume have been reported, but whether physical activity (PA) can attenuate the impact of genetic susceptibility to these traits is poorly understood. We aimed to investigate whether higher PA modify genetic impact on liver-related traits in the UK Biobank cohort. PA was self-reported, while magnetic resonance images were used to estimate liver fat (n = 27,243) and liver volume (n = 24,752). Metabolic dysfunction-associated liver disease (MASLD) and chronic liver disease (CLD) were diagnosed using ICD-9 and ICD-10 codes. Ten liver fat and eleven liver volume-associated genetic variants were selected and unweighted genetic-risk scores for liver fat (GRS_LF_) and liver volume (GRS_LV_) were computed. Linear regression analyses were performed to explore interactions between GRS_LF_/ GRS_LV_ and PA in relation to liver-related traits. Association between GRS_LF_ and liver fat was not different among lower (β = 0.063, 95% CI 0.041–0.084) versus higher PA individuals (β = 0.065, 95% CI 0.054–0.077, *p*_interaction_ = 0.62). The association between the GRS_LV_ and liver volume was not different across different PA groups (*p*_interaction_ = 0.71). Similarly, PA did not modify the effect of GRS_LF_ and GRS_LV_ on MASLD or CLD. Our findings show that physical activity and genetic susceptibility to liver-related phenotypes seem to act independently, benefiting all individuals regardless of genetic risk.

## Introduction

Metabolic dysfunction associated steatotic liver disease (MASLD) represents a spectrum of liver-related pathologies ranging from hepatic steatosis, infiltration of fat in the liver, to fibrosis, deposition of extracellular matrix products like collagen, and liver-related death^1–3^. Clinically, the presence of ≥ 5% liver fat accumulation, detected by radiological imaging, is commonly used as a threshold for initial diagnosis of MASLD^1^. Higher liver volume has also been associated with MASLD and other liver-related complications^4,5^. MASLD has become one of the major causes of chronic liver disease (CLD) in the world with an estimated global prevalence of 25%^2,3,6^.

Both liver fat content and liver volume are considered to have a heritable component^5,7^. The heritability of hepatic steatosis measured by computer tomography was previously reported to be 26–27% in three different family-based cohorts^7^. During the past several years, large scale genome-wide association studies (GWAS) have successfully identified multiple genetic variants in association with liver fat content and liver volume^5,8,9^. A recent GWAS using the UK Biobank (UKB) cohort has reported 12 genetic variants in association with liver fat content and 12 independent genetic variants in association with liver volume^5^.

Compelling evidence shows that individuals with higher physical activity (PA) have a lower risk of MASLD, liver related complications and lower liver fat content^10–14^. However, if PA can modify the impact of genetic susceptibility in the pathogenesis of MASLD and related complications is poorly understood. To our knowledge only two previous studies have examined this question. Schnurr et al. showed that higher levels of PA modified the genetic risk of MASLD using elevated alanine aminotransferase levels as a proxy for MASLD^15^. The use of serum biomarkers as a proxy for MASLD comes with several practical advantages; however, these biomarker measures can be influenced by comorbidities and are therefore not liver specific. Conversely, Ge et al. reported a null protective effect of PA on MASLD risk among individuals with high genetic risk while low sitting time or a combination of moderate PA and low sedentary time (> 4 h/day) significantly lowered the risk^16^. As such, further investigation of this topic is required. Magnetic resonance imaging proton density fat-fraction (MRI-PDFF) is a precise measure for the detection and grading of steatosis and has been widely used in epidemiological studies^17^.

Identification of interactions between lifestyle factors and genetic burden may contribute to the understanding of PA interventions in MASLD treatment and whether these interventions should be targeted based on genotype to achieve a more successful and cost-efficient intervention strategy.

In the current study, we constructed two genetic risk scores (GRSs), based on previously reported genetic loci associated with magnetic resonance imaging (MRI) derived liver fat content and liver volume, respectively. We primarily studied whether PA could attenuate the effect of these GRSs on MRI based measures of liver fat content and liver volume. Secondly, we investigated whether PA could attenuate the effect of the same GRSs on clinically diagnosed MASLD and CLD using the UKB cohort.

## Methods

### Study cohort

The UKB is a large-scale prospective study cohort (n > 500,000) with participants aged 40–69 years recruited during 2006–2010. The biobank contains extensive phenotypic and genotypic data on the participants and has been open for researchers since 2012^18^. The UKB has ethical approval from the North West Multi-Centre Research Ethics Committee (ref: 11/NW/0382) and informed written consent from all participants prior to the study. The current study was further approved by the Swedish central ethics committee (diary number 2019-03073).

A subset of the UKB cohort, including individuals with neck-to-knee MRI scans, was used when liver fat content and liver volume were considered outcomes. A larger subset of the UKB cohort including all participants with data on hospital health outcomes was included in the analyses considering MASLD and CLD as outcomes. All data-fields used in the present study are reported in Table S1.

### Measurement of liver fat and liver volume

A large-scale multi-modal imaging study (n = 100,000) is ongoing in the UKB^18^. In the current study, we included a subset of the UKB cohort (n = 32,323) who have undergone neck-to-knee MRI scans, acquired with a Siemens 1.5 T MAGNETOM Aera using a dual-echo Dixon technique, resulting in water-fat volumes covering large parts of the body. The reference measurements of liver fat content, based on proton-density fat fraction (PDFF) maps, were only available for 9,893 subjects at the time of analysis^19^. The PDFF maps were based on a single transverse slice of the liver generated with a Siemens 1.5 T MAGNETOM Aera and a three-point Dixon technique^19^.

A neural network strategy was established and trained by Langner et al.^19^. First, the neural network was trained on the neck-to-knee MR images of those with reference measurements for regression of liver fat values. After tenfold cross-validation, the trained neural network measures were applied to the rest of the neck-to-knee MRI cohort for liver fat content inference. The data generated through neural network-based approach for liver fat content measures correlated well with the reference PDFF method (R^2^ = 0.94)^19^.

A similar approach was applied to generate liver volume measures. Neck-to-knee MR images (only abdominal stations were considered) were used to computationally estimate liver volume using a deep learning approach originally established for kidney segmentation by Langner et al.^20^. Briefly, abdominal water-signal images were used in the segmentation process. Liver volume was estimated by multiplying the number of segmented voxels by the size of one voxel (liver volume = number of segmented voxels × size of 1 voxel) using 97 subjects. A segmentation model was trained on manual segmentations from these 97 subjects and was used in predicting liver volume in all neck-to-knee MRI scanned subjects. The model showed high accuracy against known manual segmentations (R^2^ = 0.86).

In the UKB, the individuals that were deemed unrelated and had passed extensive quality control were included as described by Bycroft et al.^21^. We further filtered for ethnic background and only Caucasians were included in the analyses. For the current analyses, liver fat content (n = 27,243) as well as liver volume (n = 24,752) measures were available in the UKB study participants with Caucasian ancestry.

### Genetic risk score

The data collection, genotyping and quality control in the UKB study has previously been described in detail elsewhere^21^. All UKB participants have been genotyped. Briefly, the genotyping was performed on blood samples using one of two designed arrays (UK BiLEVE Axiom Array and UK Biobank Axiom Array) that share 95% of the genetic markers. The genotypes were further imputed using the Haplotype Reference Consortium and the UK10K haplotype resource^21^. In the present study, the imputed genotypes version 3 was used.

Recently, Liu et al. performed a GWAS study (n = 32,858) in relation to abdominal MRI derived phenotypes using UKB and discovered 12 genetic variants in association with liver fat content and 12 genetic variants in association with liver volume^5^. Only independent genetic variants were included in the current study, a similar strategy has been used previously^22^. Ten liver fat content and 11 liver volume associated genetic variants were thus extracted from the UKB genetic data and were recoded based on the liver fat content/volume increasing alleles (Table S2).

Unweighted genetic risk scores for each study participant were determined by aligning trait-increasing alleles, both for liver fat content (GRS_LF_) as well as for liver volume (GRS_LV_) and summing up the total number of risk alleles, using methods that have previously been described^23^. The GRS_LF_ ranged from 2 to 18 while GRS_LV_ ranged from 0 to 14 among the study participants. For genetic risk scores, imputed genetic variants with genotype dosage < 0.5 were recoded as 0, with genotype dosage of > 0.5 to ≤ 1.50 were recoded as 1 and with genotype dosage of > 1.50 were recoded as 2. We found that two of the liver fat content associated variants were in strong LD with liver volume associated variants (rs4665985 and rs1260326, R^2^ = 0.34; rs58542926 and rs58489806, R^2^ = 0.80) and so were excluded from the GRS_LF_ and GRS_LV_ in the sensitivity analyses.

### Self-reported levels of physical activity

Questions derived from the validated International Physical Activity Questionnaire (IPAQ) short form were used to assess the weekly performance of walking, moderate PA and vigorous PA. For each type of PA, the participants were asked to estimate how many days on a typical week they spend at least 10 min doing each activity. Participants who reported an activity frequency of at least one day/week were further asked to report how many minutes on a typical day they spend doing the activity. Participants reporting a frequency of zero days/week for any of the activities were given a duration of zero.

The participants were asked to include walking at work, walking to and from work and walking for sports/leisure when estimating frequency and time of walking. For moderate and vigorous PA, the participants were asked to include activities performed for work, leisure, travel and around the house.

The obtained questionnaire data was handled according to the IPAQ guidelines^24^. Individual IPAQ scores in metabolic equivalent of task (MET)-minutes/week were calculated for each type of PA by multiplying the frequency (days/week) with the typical duration (minutes) and an activity specific MET-value. Total MET-minutes/week scores were calculated for each participant by summing up the weekly MET-minutes for walking, moderate and vigorous PA. The time variables were truncated at 180 min^24^.

Participants who answered “do not know” or “prefer not to answer” on either question were excluded. Those reporting “unable to walk”, lacking data on either frequency or duration, or with a total activity time exceeding 960 min were also excluded. Further, reported durations of less than 10 min were changed to zero^24^. After the exclusions 23,080 and 20,986 participants were included in the liver fat and liver volume cohort, respectively.

The participants were divided into three PA groups (low, moderate, and high) based on the categorical score criteria stated in the IPAQ guidelines (Text S1)^24^. All exclusions made are presented in Figs. S1 and S2.

### Other lifestyle measures

Age at baseline/recruitment was truncated to whole years. The sex of each participant was acquired from the central registry at recruitment. Both information regarding genotyping array and population substructure (first 20 genetic principal components) were obtained with the genomic UKB data. The BMI values were constructed from height and weight measured during the initial Assessment Centre visit. If either height or weight was omitted no BMI value was constructed.

The participants were asked to report their baseline smoking status (never, previous, current or prefer not to answer) in a touchscreen questionnaire. Participants were also asked to report their alcohol consumption status and estimate their current alcohol intake frequency. Individuals reporting an alcohol intake frequency of at least once or twice a week were asked to estimate an average weekly consumption of a variety of alcoholic beverages. These measures were used to estimate an average weekly alcohol intake, from which an estimated daily consumption was derived^25^. The Townsend deprivation index was calculated prior to recruitment to the UKB based on the preceding national census output areas. Each participant received a score based on the geographical area determined by their postcode. All analyses were performed as complete case analysis, the covariates were not imputed, number of individuals included in each model are reported in respective tables.

### Assessment of MASLD and chronic liver disease

Cases of MASLD and CLD were defined based on hospital health outcome codes (ICD-9 and ICD-10 codes) (Table S3). Diagnosis across all the participants hospital inpatient records were part of the dataset, including diagnoses both before and after imaging data collection i.e. prevalent and incident cases.

Self-reported cases of CLD at a nurse’s interview were also included (Table S3)^25^. If the participants were uncertain about their illness, the interviewer, a trained nurse, tried to classify it based on their description. Any illnesses that the nurse could not code were recorded as a free-text description, which was later reviewed by a doctor for coding the illness or marked it as “unclassifiable”. The MASLD/CLD cohort included n = 239,308 individuals from which n = 172 cases of MASLD and n = 371 cases of CLD were identified.

### Statistical analyses

All statistical analyses were performed using Stata (version 15, StataCorp, College Station, TX, USA). Both liver fat content and liver volume variables had skewed distributions and were transformed using rank-based inverse-normal transformation. Linear regression analyses were performed to assess the association of GRS and PA with liver fat content/volume, assuming an additive effect. Interaction analyses for GRSs and PA were performed by introducing an interaction term (GRS × PA) in the regression models, along with the main effect terms. All analyses including genotype as a variable were performed while adjusting for (a) basic model covariates: age, sex, genotyping array, and population substructure (first 20 principal components), (b) main model covariates, i.e., the basic model covariates as well as smoking status (never/previous/current), alcohol consumption (g/day), and Townsend deprivation index, and (c) main model covariates and BMI. Analyses of the effect of PA on liver fat content/liver volume (lacking genotype as a variable) were adjusted for main model covariates except genotyping array and population substructure.

To evaluate the effect of PA on GRS_LF_ and GRS_LV_ in relation to MASLD and CLD, linear regression analyses with robust standard errors were performed. We considered this the primary analysis instead of the more standard logistic regression since we want to measure interaction on an additive scale. This scale is more relevant when investigating which subgroups would benefit most from PA from a public health perspective^26^. Beta coefficients and 95% confidence intervals (CIs) limits were multiplied by 100 to express change in percentage points and the results are presented as such.

Logistic regression analyses were also performed to assess the effect of PA on GRS_LF_ and GRS_LV_ in predicting MASLD and CLD. Interaction analyses were performed by introducing an interaction term (GRS × PA) in the logistic regression models, along with the main effect terms. The analyses were adjusted according to the models listed above.

### Ethics approval statement

The UKB has ethical approval from the North West Multi-Centre Research Ethics Committee (ref: 11/NW/0382) and informed written consent from all participants prior to the study. The current study was further approved by the Swedish central ethics committee (diary number 2019-03073).

### Patient consent statement

All UK Biobank participants gave consent at recruitment.

## Results

### Characteristics of the study population

The baseline characteristics of the study participants with liver fat content (n = 23,080) and liver volume (n = 20,986) measures are reported according to the level of PA in Table 1. In both the liver fat content and the liver volume sample, more physically active groups generally had lower BMI, triglycerides, C-reactive protein, hemoglobin A1c, alkaline phosphatase and alanine aminotransferase compared to the less active groups. The most active group in both the liver fat content and the liver volume cohort had higher alcohol consumption than the less active groups. The mean liver fat percentage was below the threshold for MASLD (≥ 5%) in all three PA groups. The lowest liver volume measures were observed in the moderately active group whereas the highest liver volume measures were observed in the most active group.

**Table 1:** Characteristics of the UK Biobank participants included in the liver fat content and liver volume study populations across three levels of physical activity.

Trait	Liver fat (n = 23,080)			Liver volume (n = 20,986)		
	Level of physical activity			Level of physical activity		
	Low	Moderate	High	Low	Moderate	High
N	2855	11,120	9105	2605	10,089	8292
Age (years)	54.7 (7.2)	55.5 (7.4)	55.8 (7.6)	54.6 (7.2)	55.4 (7.4)	55.7 (7.5)
Gender, males (%)	1499 (52.50)	5442 (48.94)	4771 (52.40)	1366 (53.07)	4922 (49.23)	4339 (52.87)
Body mass index (kg/m^2^)	27.8 (4.7)	26.6 (4.2)	26.2 (3.8)	27.8 (4.7)	26.5 (4.1)	26.2 (3.8)
Waist-hip ratio	0.88 (0.09)	0.86 (0.09)	0.86 (0.08)	0.88 (0.09)	0.86 (0.09)	0.86 (0.08)
High-density lipoprotein cholesterol (mmol/L)	1.39 (0.35)	1.46 (0.37)	1.51 (0.38)	1.39 (0.35)	1.46 (0.37)	1.51 (0.38)
Low-density lipoprotein cholesterol (mmol/L)	3.64 (0.86)	3.57 (0.83)	3.56 (0.83)	3.64 (0.86)	3.58 (0.83)	3.56 (0.82)
Triglycerides (mmol/L)	1.81 (1.03)	1.66 (0.96)	1.59 (0.92)	1.81 (1.05)	1.66 (0.97)	1.59 (0.92)
C-reactive protein (mg/L)	2.36 (3.62)	2.10 (3.62)	1.84 (3.27)	2.36 (3.68)	2.08 (3.53)	1.82 (3.21)
Height (cm)	170.6 (9.4)	170.0 (9.2)	170.2 (9.1)	170.7 (9.4)	170.1 (9.2)	170.3 (9.0)
Albumin (g/L)	45.27 (2.57)	45.43 (2.54)	45.48 (2.50)	45.27 (2.60)	45.44 (2.54)	45.49 (2.49)
Alkaline phosphatase (U/L)	81.27 (23.42)	79.68 (24.50)	78.95 (22.27)	81.02 (23.01)	79.63 (24.82)	78.84 (22.05)
Alanine aminotransferase (U/L)	25.30 (17.45)	23.14 (13.26)	22.42 (12.83)	25.27 (17.45)	23.15 (13.18)	22.55 (13.14)
Aspartate aminotransferase (U/L)	25.69 (13.42)	25.38 (8.96)	26.25 (8.85)	25.63 (13.84)	25.36 (8.76)	26.30 (9.02)
Apolipoprotein A (g/L)	1.49 (0.25)	1.54 (0.26)	1.57 (0.26)	1.49 (0.25)	1.54 (0.26)	1.57 (0.26)
Apolipoprotein B (g/L)	1.05 (0.24)	1.03 (0.23)	1.02 (0.23)	1.05 (0.24)	1.03 (0.23)	1.02 (0.23)
Direct bilirubin (μmol/L)	1.84 (0.85)	1.85 (0.81)	1.88 (0.82)	1.84 (0.86)	1.86 (0.81)	1.88 (0.82)
Total bilirubin (μmol/L)	9.26 (4.64)	9.41 (4.59)	9.61 (4.64)	9.28 (4.71)	9.44 (4.57)	9.63 (4.64)
Total cholesterol (mmol/L)	5.75 (1.12)	5.71 (1.09)	5.73 (1.08)	5.74 (1.11)	5.71 (1.09)	5.73 (1.08)
Gamma-glutamyl transferase (U/L)	38.26 (42.04)	34.18 (35.41)	32.13 (30.39)	38.33 (42.23)	34.17 (34.78)	32.15 (29.83)
Glucose (mmol/L)	5.00 (1.19)	5.00 (0.98)	4.98 (0.90)	5.00 (1.22)	5.00 (0.96)	4.98 (0.88)
Hemoglobin A1c (mmol/mol)	35.12 (5.55)	35.00 (5.31)	34.75 (4.57)	35.13 (5.63)	34.97 (5.27)	34.71 (4.53)
Lipoprotein A (nmol/L)	44.71 (49.56)	44.65 (49.82)	43.77 (49.22)	44.64 (49.51)	44.71 (49.89)	43.95 (49.32)
Total protein (g/L)	72.20 (3.81)	72.22 (3.95)	72.07 (3.94)	72.16 (3.79)	72.22 (3.95)	72.08 (3.94)
Alcohol consumption (g/day)	22.63 (17.92)	22.18 (17.11)	23.50 (18.29)	22.87 (18.13)	22.27 (17.12)	23.66 (18.43)
Smoking status (%)						
Never	1731 (60.6)	6922 (62.2)	5370 (59.0)	1582	6282	4866
Previous	931 (32.6)	3530 (31.7)	3191 (35.0)	844	3204	2920
Current	189 (6.6)	653 (5.9)	531 (5.8)	175	588	495
Townsend deprivation index						
Liver fat (%)	4.64 (4.81)	3.94 (4.23)	3.60 (3.91)			
Liver volume (cm^3^)				1466.44 (330.50)	1409.74 (295.87)	1427.72 (291.51)
Liver fat GRS	10.40 (1.94)	10.43 (1.90)	10.36 (1.94)			
Liver volume GRS				6.11 (1.78)	6.07 (1.82)	6.07 (1.84)

Data is presented as mean (standard deviation) or otherwise specified.

### Genetic predisposition and physical activity in relation to liver fat content and liver volume

Among individuals with liver fat content (n = 23,080) measures, GRS_LF_ was positively associated with liver fat content (β = 0.058, 95% CI 0.052–0.065, *p*-value < 0.001) after adjusting for age, sex, genotyping array, and population substructure. The GRS_LF_ explained 5.3% (R^2^ = 0.053) of the liver fat content phenotypic variance. Higher PA was associated with lower liver fat content (β =  − 0.143, 95% CI − 0.164 to − 0.121, *p*-value < 0.001) when the regression model was adjusted for main model covariates except genotyping array and population substructure.

**Figure 1:**
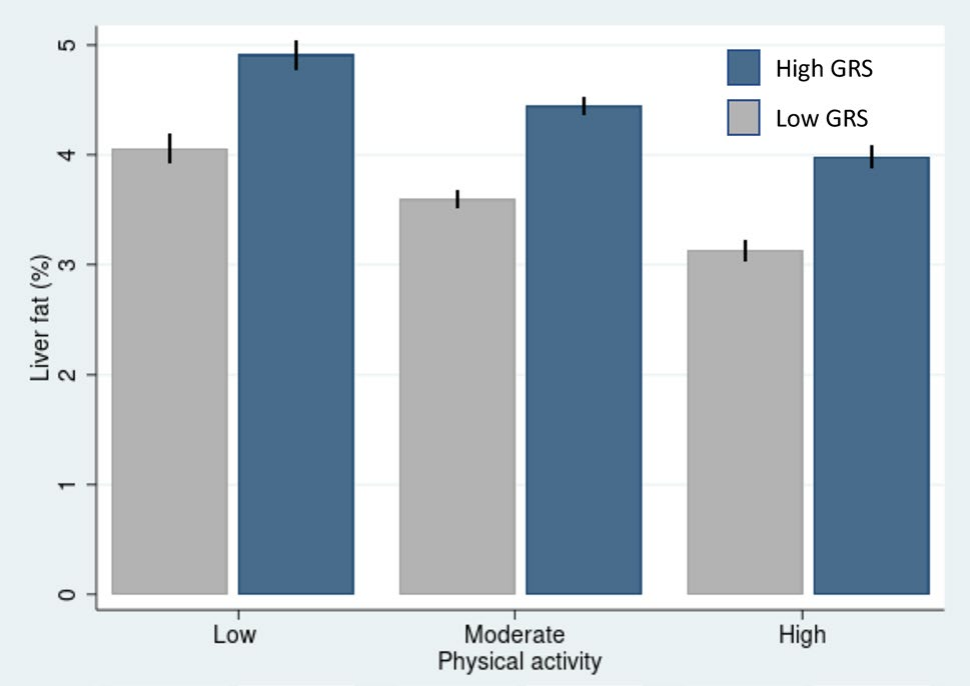
Association between the liver fat associated genetic risk score (GRS_LF_) and liver fat content across three levels of physical activity. The participants were divided into low and high genetic risk based on the median value (Median = 10.016). The two GRS groups were significantly associated with liver fat content (*p*-value < 0.001). The error bars represent 95% CIs.

In the regression model adjusted for main model covariates, higher GRS_LF_ was associated with slightly higher liver fat content among individuals in each of the three PA groups (Low PA (β = 0.063, 95% CI 0.041–0.084, *p*-value < 0.001); moderate PA (β = 0.059, 95% CI 0.048–0.069, *p*-value < 0.001); high PA (β = 0.065, 95% CI 0.054–0.077, *p*-value < 0.001)). However, we did not observe significant interaction between GRS_LF_ and PA in relation to liver fat content (*p*_interaction_ = 0.62). Similar results were observed when analyses were adjusted for basic (*p*_interaction_ = 0.95) and main + BMI model covariates (*p*_interaction_ = 0.91) (Table 2, Fig. 1).

**Table 2:** Association between GRS_LF_ and liver fat content across different levels of PA (n = 23,080).

Model	Level of physical activity						*p*_interaction_
	Low (n = 2855)		Moderate (n = 11,120)		High (n = 9105)		
	Beta (95% CI)	*p*-value	Beta (95% CI)	*p*-value	Beta (95% CI)	*p*-value	
Basic	0.061 (0.042; 0.080)	< 0.001	0.056 (0.047; 0.066)	< 0.001	0.058 (0.048; 0.069)	< 0.001	0.95
Main	0.063 (0.041; 0.084)	< 0.001	0.059 (0.048; 0.069)	< 0.001	0.065 (0.054; 0.077)	< 0.001	0.62
Main + BMI	0.062 (0.043; 0.082)	< 0.001	0.057 (0.048; 0.067)	< 0.001	0.061 (0.051;0.072)	< 0.001	0.91

Basic model (n = 23,080) covariates include genotyping array, first 20 genetic principal components, age, and sex. Main model (n = 18,057) covariates include basic model covariates + alcohol consumption (g/day), smoking status and Townsend deprivation index. Main + BMI model (n = 18,057) covariates include those of the main model and body mass index (BMI).

*GRS*_*LF*_ liver fat associated genetic risk score.

In the sensitivity analysis, similar results were observed after excluding liver fat content associated genetic variants which were in LD with liver volume associated genetic variants (Table S4).

In the liver volume cohort (n = 20,986), higher GRS_LV_ was associated with higher liver volume (β = 0.066, 95% CI 0.059–0.073, *p*-value < 0.001) after adjusting for age, sex, genotyping array, and population substructure. The GRS_LV_ explained 1.6% (R^2^ = 0.016) of the phenotypic variance in the liver volume trait. Physical activity was not associated with liver volume (β = -0.018, 95% CI − 0.040 to 0.004, *p*-value = 0.12) when the analyses were adjusted for main model covariates except genotyping array and population substructure.

**Figure 2:**
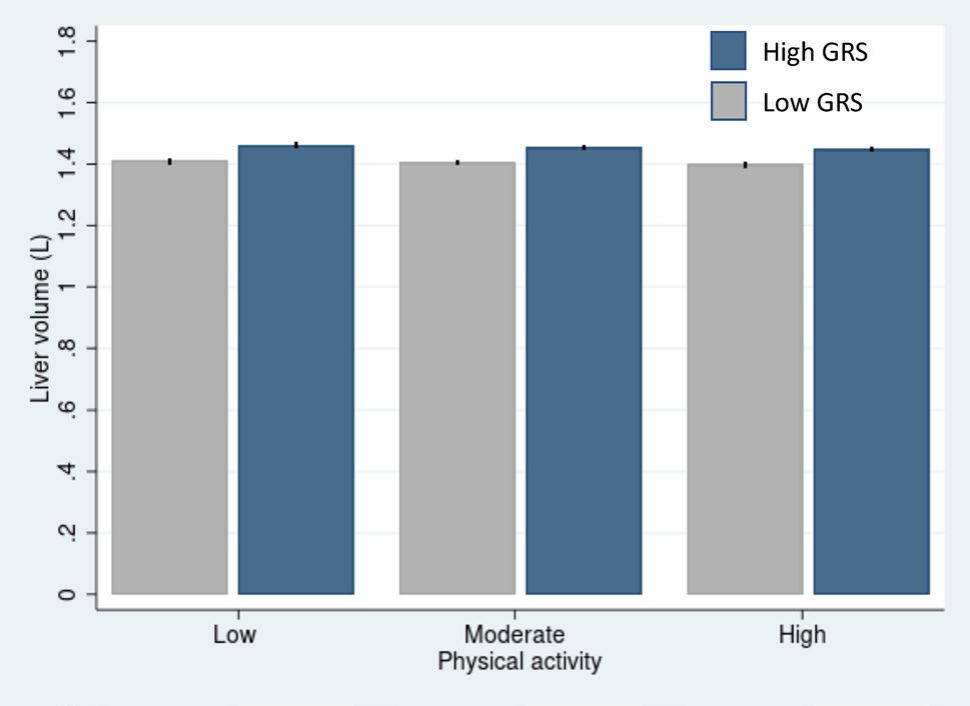
Association between the liver volume associated genetic risk score (GRS_LV_) and liver volume across three levels of physical activity. The participants were divided into low and high genetic risk based on the median value (Median = 6.0). The two GRS groups were significantly associated with liver fat content (*p*-value < 0.001). Error bars represent 95% CIs.

After adjustment for main model covariates, higher GRS_LV_ was associated with slightly higher liver volume among individuals in each of the three PA groups (Low PA (β = 0.066, 95% CI 0.040–0.092, *p*-value < 0.001); moderate PA (β = 0.063, 95% CI 0.051–0.074, *p*-value < 0.001); high PA (β = 0.067, 95% CI 0.055–0.079, *p*-value < 0.001)). However, the interaction estimate was not significant (*p*_interaction_ = 0.71). Similar results were observed when the regression models were adjusted for basic model covariates (*p*_interaction_ = 0.76) and main + BMI (*p*_interaction_ = 0.44) (Table 3, Fig. 2).

**Table 3:** Association between GRS_LV_ and liver volume across different levels of PA (n = 20,986).

Model	Level of physical activity						*p*_interaction_
	Low (n = 2605)		Moderate (n = 10,089)		High (n = 8292)		
	Beta (95% CI)	*p*-value	Beta (95% CI)	*p*-value	Beta (95% CI)	*p*-value	
Basic	0.068 (0.045; 0.092)	< 0.001	0.066 (0.055; 0.076)	< 0.001	0.065 (0.054; 0.076)	< 0.001	0.76
Main	0.066 (0.040; 0.092)	< 0.001	0.063 (0.051; 0.074)	< 0.001	0.067 (0.055; 0.079)	< 0.001	0.71
Main + BMI	0.050 (0.028; 0.072)	< 0.001	0.056 (0.046; 0.066)	< 0.001	0.059 (0.048; 0.069)	< 0.001	0.44

Basic model (n = 20,986) covariates include genotyping array, first 20 genetic principal components, age, and sex. Main model (n = 16,466) covariates include basic model covariates + alcohol consumption (g/day), smoking status and Townsend deprivation index. Main + BMI model (n = 16,305) covariates include those of the main model and body mass index (BMI).

*GRS*_*LV*_ liver volume associated genetic risk score.

In the sensitivity analysis, excluding liver volume associated genetic variants which were in LD with liver fat content associated genetic variants, the results remained materially similar (Table S5).

### Genetic predisposition, physical activity in relation to MASLD and CLD

The prevalence of MASLD and CLD in the study population was 0.07% (172/239,308) and 0.16% (371/239,308), respectively. Higher GRS_LF_ was associated with higher odds of both MASLD (OR = 1.17, 95% CI 1.08–1.27, *p*-value < 0.001) and CLD (OR = 1.18, 95% CI 1.11–1.24, *p*-value < 0.001) when adjusting for age, sex, genotyping array, and population substructure. Higher PA was associated with lower odds for both traits (MASLD: OR = 0.68, 95% CI 0.51–0.91, *p*-value < 0.009; CLD: OR = 0.70, 95% CI 0.57–0.86, *p*-value = 0.001) after adjustment for main model covariates except genotyping array and population substructure.

When adjusting the linear regression with robust standard errors analyses for main model covariates, individuals in the high PA group with higher GRS_LF_ had a slightly higher likelihood of having a MASLD diagnosis (β = 0.012, 95% CI 0.003–0.021, *p*-value = 0.008), while we could not detect an association in the moderate (β = 0.008, 95% CI − 0.004 to 0.020, *p*-value = 0.211) and low (β = 0.004, 95% CI − 0.012 to 0.021, *p*-value = 0.626) PA groups. However, the interaction was not significant (*p*_interaction_ = 0.36) (Table 4). Similarly, GRS_LF_ was associated with higher odds of MASLD among individuals in the high PA group (OR = 1.41, 95% CI 1.15–1.73, *p*-value = 0.001), but not in the moderate (OR = 1.11, 95% CI 0.97–1.27, *p*-value = 0.119) and low (OR = 1.07, 95% CI 0.80–1.42, *p*-value = 0.669) PA groups. Nonetheless, the interaction in the logistic regression analysis was not statistically significant (*p*_interaction_ = 0.09) (Table S6).

**Table 4:** The association of liver fat content associated genetic variants with MASLD and CLD (N = 239,308).

Outcome	Physical activity						
	Low (n = 28,253)		Moderate (n = 112,364)		High (n = 98,691)		*p*_interaction_
	Beta (95% CI)*	*p*-value	Beta (95% CI)*	*p*-value	Beta (95% CI)*	*p*-value	
MASLD (n_cases_ = 172)							
Basic	0.012 (− 0.006; 0.030)	0.198	0.011 (0.001; 0.021)	0.036	0.012 (0.004; 0.020)	0.005	0.96
Main	0.004 (− 0.012; 0.021)	0.626	0.008 (− 0.004; 0.020)	0.211	0.012 (0.003; 0.021)	0.008	0.36
Main + BMI	0.004 (− 0.013; 0.020)	0.649	0.008 (− 0.004;0.020)	0.212	0.012 (0.003;0.021)	0.009	0.36
CLD (n_cases_ = 371)							
Basic	0.044 (0.006; 0.082)	0.023	0.027 (0.014; 0.040)	< 0.001	0.016 (0.004; 0.029)	0.008	0.11
Main	0.035 (− 0.001 ;0.070)	0.053	0.016 (0.002; 0.029)	0.023	0.015 (0.004; 0.027)	0.009	0.39
Main + BMI	0.035 (− 0.001 ;0.070)	0.054	0.016 (0.002;0.029)	0.023	0.015 (0.004 ;0.027)	0.009	0.39

The basic model (n = 239,308) was adjusted for age, sex, first 20 genetic principal components and genotyping array. Main model (n = 173,387) covariates include basic model covariates + alcohol consumption (g/day), smoking status and Townsend deprivation index. Main + BMI model (n = 173,029) covariates include those of the main model and body mass index (BMI). MASLD; metabolic dysfunction associated steatotic liver disease, CLD; chronic liver disease.

*Betas and 95% CI limits were multiplied by 100 and reported as percentage.

Higher GRS_LF_ was associated with a higher likelihood for CLD in all three PA groups. Although the strength of association appeared greater in individuals in the low PA group the interaction results were not statistically significant in any of the adjusted models (Table 4). When these analyses were repeated with logistic regression, GRS_LF_ was not significantly associated with higher odds of MASLD (*p*_interaction_ = 0.09) or CLD (*p*_interaction_ = 0.91) when adjusting for main model covariates (Table S6).

A higher GRS_LV_ was associated with higher odds of MASLD (OR = 1.14, 95% CI 1.05–1.24, *p*-value = 0.001) but not CLD (OR = 1.04, 95% CI 0.98–1.10, *p*-value = 0.194) when adjusting for age, sex, genotyping array, and population substructure. However, no associations between GRS_LV_ and MASLD or CLD were detected among any of the PA groups when adjusting for main model covariates, and the interaction terms were not significant. This was the case for analyses based on linear regression with robust standard errors (Table 5) and analyses based on logistic regression (Table S7).

**Table 5:** The association of liver volume associated genetic variants with MASLD and CLD (n = 239,308).

Outcome	Physical activity						
	Low (n = 28,253)		Moderate (n = 112,364)		High (n = 98,691)		*p*_interaction_
	Beta (95% CI)*	*p*-value	Beta (95% CI)*	*p*-value	Beta (95% CI)*	*p*-value	
MASLD (n_cases_ = 172)							
Basic	0.004 (− 0.015; 0.022)	0.707	0.012 (0.002; 0.022)	0.023	0.009 (0.0001; 0.018)	0.047	0.84
Main	0.010 (− 0.011; 0.032)	0.346	0.007 (− 0.003; 0.018)	0.180	0.006 (− 0.002; 0.013)	0.138	0.65
Main + BMI	0.010 (− 0.011; 0.031)	0.353	0.007 (− 0.004; 0.018)	0.195	0.005 (− 0.002; 0.013)	0.147	0.65
CLD (n_cases_ = 371)							
Basic	0.024 (− 0.009; 0.057)	0.149	0.002 (− 0.011;0.016)	0.718	0.005 (− 0.006; 0.015)	0.387	0.44
Main	− 0.007 (− 0.037 ;0.023)	0.659	− 0.001 (− 0.015; 0.014)	0.911	0.004 (− 0.006 ;0.013)	0.421	0.36
Main + BMI	− 0.007 (− 0.037; 0.023)	0.654	− 0.001 (− 0.015; 0.013)	0.888	0.004 (− 0.006; 0.013)	0.425	0.36

The basic model (n = 239,308) was adjusted for age, sex, first 20 genetic principal components and genotyping array. Main model (n = 173,387) covariates include basic model covariates + alcohol consumption (g/day), smoking status and Townsend deprivation index. Main + BMI model (n = 173,029) covariates include those of the main model and body mass index (BMI). MASLD; metabolic dysfunction associated steatotic liver disease, CLD; chronic liver disease.

*Betas and 95% CI limits were multiplied by 100 and reported as percentage.

In the sensitivity analysis, we excluded two pairs of genetic variants (rs4665985 and rs1260326, rs58542926 and rs58489806) that were in LD for liver fat and liver volume-associated traits. We found that the results remained materially similar in all analyses (Tables S8–S11).

## Discussion

To our knowledge, this is the first large-scale epidemiological study investigating interactions between PA and genetic predisposition to objectively measured liver fat content and liver volume in predicting respective traits. We found that the genetic effects on these traits were not modified by PA. Furthermore, we observed that PA did not modify the impact of liver associated genetic predisposition on the likelihood of having a MASLD or CLD diagnosis.

A previous study has demonstrated that higher PA can mitigate the impact of genetic predisposition to higher alanine aminotransferase (ALT) levels on measured ALT, which served as a proxy for MASLD^15^. In contrast, we examined MRI-derived measurements of liver fat content and liver volume inferred through a neural network-based approach instead of ALT levels. While serum biomarkers like ALT are practical for large-scale epidemiological studies due to their routine assessments and good inter-laboratory reproducibility, they have certain limitations. For example, they lack specificity for the liver and adjusting for comorbid conditions leading to elevated ALT levels can be complex^17,27^. Further, around 19% of European MASLD patients have normal ALT values, so, a large proportion of MASLD cases are likely excluded by using ALT levels as a proxy for MASLD^28^.

The non-invasive MRI-PDFF measures have been shown to correlate well with gold-standard histology-determined steatosis in patients with MASLD (*p*-value > 0.0001)^29^. By utilizing deep learning, a larger population than those with MRI-PDFF measures in the UKB could be included in our study^19,30^. To further enhance the clinical relevance of our study, we also examined diagnoses of MASLD and CLD, as determined by healthcare professionals at hospitals. Despite its clinical relevance, relying on diagnoses set by healthcare professionals may exclude cases of MASLD and CLD, as not all individuals seek medical care. Furthermore, the healthy volunteer bias in cohorts like UKB comprising the generalizability in addition to lack of external validity is well known^31^. The healthy volunteer bias likely contributed to the substantially lower prevalence of MASLD and CLD in our cohort compared to the general population. This bias also resulted in generally healthy liver fat content and liver volume study populations, based on biomarker characteristics.

Large sample sizes are needed to identify interactions, especially if the effect estimates are of low magnitude^32,33^. However, the beta estimates presented here were similar across groups, indicating no clinically significant interactions being present. The only exception to this was individuals in the high PA group having higher odds for MASLD compared to those in the moderate and low PA groups. This could be due to reverse causation, the limitation of including both prevalent and incident cases. Individuals who increase their PA after diagnosis could cause overinflation of odds in the high PA group. In this scenario, the higher odds ratios do not reflect a true elevation of risk as the cases are prevalent. A recent prospective study demonstrated that PA did not attenuated the genetic effect in relation to MASLD^16^. PA was assessed similarly as in our study using IPAQ while PA groups were constructed based on MET values and not IPAQ categorical score criteria as in the present study. Even though, only incident cases were included and the frequency of those were more in line with the expected no significant interactions between PA and genetic risk were identified among individuals with high genetic risk^16^. Therefore, further larger studies with incident cases of MASLD and CLD are required to examine the potential effect of PA on genetic risk for these outcomes in more depth. Future studies should also explore interactions between liver-associated genetic risk and PA in the more severe and progressive forms of MASLD namely, metabolic dysfunction associated steatohepatitis (MASH) and MASH with fibrosis.

Our results suggest that PA interventions should be recommended to everyone with MASLD, given that we observed that higher physical activity has a protective effect in relation to liver fat content, liver volume, MASLD and CLD, in line with previous studies. However, targeting PA interventions based on current liver associated genetic information does not appear to increase efficiency as no interaction effects were identified.

Strengths of the present study include deeply phenotyped liver fat content and liver volume measures inferred through deep learning, which were strongly correlated with the UKB based MRI reference measures^19^. The categories of PA we used are beneficial for interpretation of the results, since the most active group reaches the recommendations for health enhancing PA^24,34^. Furthermore, we considered potential confounding factors including alcohol consumption, BMI, and material deprivation (Townsend deprivation index). The genetic variants used to construct the genetic risk scores (GRS_LF_ and GRS_LV_) were reported to associate with liver fat content and liver volume, respectively, in a previous GWAS^5^. Several of the genetic variants used are shared between studies with reported associations with MASLD, liver fat, liver disorders and metabolic traits, supporting our use of these genetic variants^7,8,35–39^. Since the original GWAS was conducted within the UKB data there was a risk that effect estimates for the genetic component would be overestimated, compared with an independent sample. We therefore created unweighted genetic risk scores to reduce this potential overestimation of effects.

Our study also had several limitations. Physical activity assessment by wrist-worn accelerometer data is available for approximately 100,000 UKB participants, to date. Unfortunately, the accelerometer cohort did not substantially overlap with the liver fat content and liver volume study samples. As such, we decided to use questionnaire-based measures of PA to improve statistical power. The IPAQ short form has previously showed acceptable results for both reliability and validity^40,41^. However, self-reported measures of PA are subjective and prone to measurement errors, including misreporting or cognitive difficulties recalling performed activities^41^. As being physically active is recommended and to some extent socially rewarded, less active individuals might report higher levels of activity than they in fact performed. Misreporting due to cognitive difficulties should on the other hand be more random.

While the UKB participants were recruited during 2006–2010, the imaging study did not start until 2014, meaning that the baseline PA data used in the present study was collected some years before the participants underwent the radiological examinations. As the PA and MRI-PDFF measures were not collected simultaneously, changes in behavior or liver health might have occurred between the assessment periods. However, the advantage is the reduced risk of reverse causality.

In conclusion, we did not observe that PA modified the effect of genetic predisposition on elevated liver fat content and liver volume, measured without a human operator. Our results suggest that targeting PA interventions by using current knowledge of liver associated genetic risk is not likely to improve public health nor offer a route to more cost-efficient healthcare.

### Supplementary Information

Supplementary Information.

## Data Availability

The study utilized data sourced from the UK Biobank Cohort, which requires successful registration and application for access. Further information on accessing the UK Biobank data source can be found on their website: https://www.ukbiobank.ac.uk/.

## Abbreviations

ALT: Alanine amoinotransferase
BMI: Body mass index
CLD: Chronic liver disease
GRS: Genetic risk score
GRS_LF_: Liver fat associated genetic risk score
GRS_LV_: Liver volume associated genetic risk score
GWAS: Genome-wide association study
ICD: International classification of diseases
IPAQ: International physical activity questionnaire
MASLD: Metabolic dysfunction associated steatotic liver disease
MET: Metabolic equivalent of task
MRI: Magnetic resonance imaging
MRI-PDFF: Magnetic resonance imaging proton density fat-fraction
PA: Physical activity
PDFF: Proton density fat-fraction
UKB: UK Biobank
