# Supplementary material for: The interaction of genetics and physical activity in the pathogenesis of metabolic dysfunction associated liver disease
Source: Sci Rep. 2024 Aug 1;14:17817. doi: 10.1038/s41598-024-68271-4 (PMC11294342; doi:10.1038/s41598-024-68271-4)
Supplement: Supplementary file 1 — Supplementary Information. [file 41598_2024_68271_MOESM1_ESM.docx]

**The interaction of genetics and physical activity in the pathogenesis of metabolic dysfunction associated liver disease**

Hanna Frostdahl, Nouman Ahmad, Ulf Hammar, Andrés Martínez Mora, Taro Langner, Tove Fall, Joel Kullberg, Håkan Ahlström, Hannah L. Brooke, Shafqat Ahmad

Additional file 1:

[Supplementary text 1. Division into groups based on level of physical activity. Participants were categorized into three levels of physical activity, following the guidelines of International Physical Activity Questionnaire short form. 3](#_Toc153449240)

[Table S1. UK Biobank data-fields used 4](#_Toc153449241)

[Table S2. Summary of the liver fat and liver volume genetic variants 5](#_Toc153449242)

[Table S3. The ICD-9 and ICD-10 codes used to classify cases of metabolic dysfunction associated steatotic liver disease (MASLD) and chronic liver disease (CLD). 6](#_Toc153449243)

[Table S4. Sensitivity analysis of association between GRSLF and liver fat content across different levels of PA 7](#_Toc153449244)

[Table S5. Sensitivity analysis of association between GRSLV and liver volume across different levels of PA 8](#_Toc153449245)

[Table S6. Multivariable logistic regression analyses of liver fat content associated genetic variants in predicting metabolic dysfunction associated steatotic liver disease (MASLD) and chronic liver disease (CLD) 9](#_Toc153449246)

[Table S7. Multivariable logistic regression analyses of liver volume associated genetic variants in predicting metabolic dysfunction associated steatotic liver disease (MASLD) and chronic liver disease (CLD) 10](#_Toc153449247)

[Table S8. Sensitivity linear regression with robust standard errors analyses of liver fat content associated genetic variants in relation to metabolic dysfunction associated steatotic liver disease (MASLD) and chronic liver disease (CLD) 11](#_Toc153449248)

[Table S9. Sensitivity linear regression with robust standard errors analyses of liver volume associated genetic variants in relation to metabolic dysfunction associated steatotic liver disease (MASLD) and chronic liver disease (CLD) 12](#_Toc153449249)

[Table S10. Sensitivity multivariable logistic regression analyses of liver fat content associated genetic variants in predicting metabolic dysfunction associated steatotic liver disease (MASLD) and chronic liver disease (CLD) 13](#_Toc153449250)

[Table S11. Sensitivity multivariable logistic regression analyses of liver volume associated genetic variants in predicting metabolic dysfunction associated steatotic liver disease (MASLD) and chronic liver disease (CLD) 14](#_Toc153449251)

[Figure S1. Exclusions made in the liver fat cohort based on answers to the International Physical Activity Questionnaire. 15](#_Toc153449252)

[Figure S2. Exclusions made in the liver volume cohort based on answers to the International Physical Activity Questionnaire. 16](#_Toc153449253)

# **Supplementary text 1.** Division into groups based on level of physical activity. Participants were categorized into three levels of physical activity, following the guidelines of International Physical Activity Questionnaire short form (1).

**Division into PA groups was performed according to the categorical score criteria:**

***Low***

Individuals not meeting the criteria for the “moderate” or “high” activity levels were grouped into the least active group.

***Moderate***

Participants were classified as moderately active if meeting at least one of the following criteria:

- At least 3 days of vigorous-intensity activities of ≥20 minutes per day
- At least 5 days of moderate-intensity activity and/or walking of ≥30 minutes per day
- At least 5 days of any combination of walking, moderate- and vigorous-intensity activities achieving a total PA of at least 600 MET-minutes/week

***High***

Participants were classified “high” if meeting at least one of the following criteria:

- At least 3 days of vigorous-intensity activities and additionally achieving a total PA of least 1500 MET-minutes/week
- Achieving at least 3000 MET-minutes/week of any combination of walking, moderate- and vigorous-intensity activities performed during at least 7 days

# **Table S1**. UK Biobank data-fields used.

| **Data-field** | **Description** |
| --- | --- |
| 20201-2.0 | Neck-to-knee MRI scans |
| 22436 | Reference measurements of liver fat based on PDFF maps |
| 22006 | Genetic ethnic grouping |
| 22020 | Used in genetic principal components |
| 21022 | Age at baseline |
| 31 | Sex |
| 21001 | Body mass index |
| 22000 | Genotyping array |
| 22009 | Genetic principal components |
| 20116 | Smoking status |
| 20117 | Alcohol drinking status |
| 1558 | Alcohol intake frequency |
| 1568, 1578, 1588, 1598 and 1608 | Alcoholic beverages |
| 22189 | Townsend deprivation index |
| 864 | Number of days/week walked 10+ minutes |
| 874 | Duration of walks |
| 884 | Number of days/week of moderate PA 10+ minutes |
| 894 | Duration of moderate PA |
| 904 | Number of days/week of vigorous PA 10+ minutes |
| 914 | Duration of vigorous PA |
| 41202 | ICD-10 codes (main diagnosis) |
| 41204 | ICD-10 codes (secondary diagnosis) |
| 41203 | ICD-9 codes (main diagnosis) |
| 41205 | ICD-9 codes (secondary diagnosis) |
| 20002 | Diagnoses self-reported at nurse's interview |

MRI; magnetic resonance imaging, PDFF; proton-density fat fraction, PA; physical activity.

**Table S2**. Summary of the liver fat and liver volume genetic variants.

| **SNP (rs_id)** | **Trait** | **Chr: position** | **Nearby gene** | **Beta** | ***p*-value** | **Effect allele** | **Other allele** | **EAF** |
| --- | --- | --- | --- | --- | --- | --- | --- | --- |
| rs2642438 | Liver fat | 1: 220970028 | *MARC1* | 0.05 | <0.001 | G | A | 0.71 |
| rs4665985 | Liver fat | 2: 27753878 | *C2orf16* | 0.04 | <0.001 | C | A | 0.27 |
| 4: 100472229 | Liver fat | 4: 100472229 | *MTTP* | 0.03 | 0.001 | GATTTATAGTTCAGAGA | G | 0.74 |
| rs112875651 | Liver fat | 8: 126506694 | *TRIB1* | 0.03 | 0.001 | G | A | 0.62 |
| rs7029757 | Liver fat | 9: 132566666 | *TOR1B* | 0.04 | 0.001 | G | A | 0.91 |
| rs11446981 | Liver fat | 10: 113950257 | *GPAM* | 0.01 | 0.5 | T | TA | 0.30 |
| rs55714539 | Liver fat | 19: 18207397 | *MAST3* | 0.03 | <0.001 | C | A | 0.34 |
| rs58542926 | Liver fat | 19: 19379549 | *TM6SF2* | 0.28 | <0.001 | T | C | 0.07 |
| rs429358 | Liver fat | 19: 45411941 | *APOE* | 0.09 | <0.001 | T | C | 0.85 |
| rs738409 | Liver fat | 22: 44324727 | *PNPLA3* | 0.19 | <0.001 | G | C | 0.22 |
| rs193084249 | Liver volume | 1: 26987646 | ARID1A | 0.18 | <0.001 | G | A | 0.02 |
| rs1260326 | Liver volume | 2: 27730940 | GCKR | 0.07 | <0.001 | T | C | 0.39 |
| rs79287178 | Liver volume | 3: 172294500 | TNFSF10 | 0.18 | <0.001 | A | G | 0.03 |
| rs1009064 | Liver volume | 6: 52623490 | GSTA2 | 0.04 | <0.001 | G | C | 0.35 |
| rs753444559 | Liver volume | 6: 127066487 | RSPO3 | 0.05 | <0.001 | G | GA | 0.51 |
| rs4240624 | Liver volume | 8: 9184231 | PPP1R3B | 0.18 | <0.001 | G | A | 0.09 |
| rs7896518 | Liver volume | 10: 65104500 | REEP3 | 0.05 | <0.001 | A | G | 0.57 |
| rs10881959 | Liver volume | 10: 93507964 | TNKS2 | 0.05 | <0.001 | T | G | 0.58 |
| rs139974673 | Liver volume | 15: 44027885 | PDIA3 | 0.25 | <0.001 | C | T | 0.03 |
| 16:53812783 | Liver volume | 16:53812783 |  | 0.05 | <0.001 | T | TTTTG | 0.39 |
| rs58489806 | Liver volume | 19: 19456917 | MAU2 | 0.08 | <0.001 | T | C | 0.09 |

Chr: chromosome, EAF: effect allele frequency. The variants are previously reported by Liu *et al*. (2).

# **Table S3**. The ICD-9 and ICD-10 codes used to classify cases of metabolic dysfunction associated steatotic liver disease (MASLD) and chronic liver disease (CLD).

| **Traits** | **ICD-10** | **ICD-9** |
| --- | --- | --- |
| **MASLD** | K760 |  |
| **CLD** | K702; K703; K704; K717; K721; K74; K740; K741; K742; K743; K744; K745; K746 | 27103; 4562; 571; 5712; 5715; 57150; 57151; 57158; 57159; 5716 |

Cases of MASLD and CLD were defined based on diagnosis in hospital inpatient records. Self-reported cases at a nurse’s interview were also included in the dataset used in the logistic regression analyses.

**Table S4**. Sensitivity analysis of association between GRSLF and liver fat content across different levels of PA (n=23,080).

|  | **Level of Physical Activity** | | | | | |  |
| --- | --- | --- | --- | --- | --- | --- | --- |
|  | Low (n=2,855) | | Moderate (n=11,120) | | High (n=9,105) | |  |
| Model | Beta (95% CI) | *p*-value | Beta (95% CI) | *p*-value | Beta (95% CI) | *p*-value | *p*_interaction_ |
|  |  |  |  |  |  |  |  |
| Basic | 0.052 (0.031;0.072) | <0.001 | 0.048 (0.038;0.058) | <0.001 | 0.051 (0.040;0.062) | <0.001 | 0.99 |
|  |  |  |  |  |  |  |  |
| Main | 0.053 (0.029;0.076) | <0.001 | 0.052 (0.040;0.064) | <0.001 | 0.058 (0.045;0.071) | <0.001 | 0.57 |
|  |  |  |  |  |  |  |  |
| Main+BMI | 0.053 (0.031;0.074) | <0.001 | 0.049 (0.039;0.060) | <0.001 | 0.056 (0.044;0.067) | <0.001 | 0.59 |
|  |  |  |  |  |  |  |  |

The liver fat associated genetic risk score (GRS_LF_) used in the sensitivity analysis was constructed excluding rs4665985 and rs58542926 as these variants were in linkage disequilibrium with liver volume associated variants rs1260326 (R^2^=0.34) and rs58489806 (R^2^=0.80), respectively. The basic model (n=23,080) covariates were genotyping array, first 20 genetic principal components, age and sex. Main model (18,057) covariates were the basic model covariates + alcohol consumption (g/day), smoking status and Townsend deprivation index. The main+BMI model (18,057) was adjusted for the main model covariates and body mass index (BMI).

**Table S5**. Sensitivity analysis of association between GRSLV and liver volume across different levels of PA (n=20,986).

|  | **Level of Physical Activity** | | | | | |  |
| --- | --- | --- | --- | --- | --- | --- | --- |
|  | **Low (n=2,605)** | | **Moderate (n=10,089)** | | **High (n=8,292)** | |  |
| Model | Beta (95% CI) | *p*-value | Beta (95% CI) | *p*-value | Beta (95% CI) | *p*-value | *p*_interaction_ |
|  |  |  |  |  |  |  |  |
| Basic | 0.067 (0.041;0.093) | <0.001 | 0.065 (0.053;0.077) | <0.001 | 0.060 (0.048;0.072) | <0.001 | 0.47 |
|  |  |  |  |  |  |  |  |
| Main | 0.060 (0.032;0.089) | <0.001 | 0.061 (0.048;0.074) | <0.001 | 0.060 (0.047;0.073) | <0.001 | 0.90 |
|  |  |  |  |  |  |  |  |
| Main+BMI | 0.040 (0.016;0.064) | 0.001 | 0.051 (0.040;0.062) | <0.001 | 0.051 (0.040;0.063) | <0.001 | 0.49 |
|  |  |  |  |  |  |  |  |

The liver volume associated genetic risk score (GRS_LV_) used in the sensitivity analysis was constructed excluding rs1260326 and rs58489806 as these variants were in linkage disequilibrium with liver volume associated variants rs4665985 (R^2^=0.34) and rs58542926 (R^2^=0.80), respectively. The basic model (n=20,986) covariates were genotyping array, first 20 genetic principal components, age and sex. Main model (n=16,466) covariates were the basic model covariates + alcohol consumption (g/day), smoking status and Townsend deprivation index. The main+BMI model (n=16,305) was adjusted for the main model covariates and body mass index (BMI).

**Table S6**. Multivariable logistic regression analyses of liver fat content associated genetic variants in predicting metabolic dysfunction associated steatotic liver disease (MASLD) and chronic liver disease (CLD) (n=239,308).

|  |  | **Physical Activity** | | | | | | |
| --- | --- | --- | --- | --- | --- | --- | --- | --- |
|  |  | Low (n=28,253) | | Moderate (n=112,364) | | High (n=98,691) | |  |
| Outcome | | OR (95% CI) | *p*-value | OR (95% CI) | *p*-value | OR (95% CI) | *p*-value | *p*_interaction_ |
|  | |  |  |  |  |  |  |  |
| MASLD (n_cases_=172) | |  |  |  |  |  |  |  |
| Basic | | 1.14 (0.93;1.40) | 0.201 | 1.14 (1.02;1.27) | 0.017 | 1.25 (1.08;1.44) | 0.002 | 0.42 |
| Main | | 1.07 (0.80;1.42) | 0.669 | 1.11 (0.97;1.27) | 0.119 | 1.41 (1.15;1.73) | 0.001 | 0.09 |
| Main+BMI | | 1.06 (0.79;1.42) | 0.702 | 1.11 (0.97;1.27) | 0.131 | 1.42 (1.16;1.75) | 0.001 | 0.10 |
|  | |  |  |  |  |  |  |  |
| CLD (n_cases_=371) | |  |  |  |  |  |  |  |
| Basic | | 1.19 (1.05;1.34) | 0.006 | 1.18 (1.09;1.28) | <0.001 | 1.16 (1.05;1.27) | 0.004 | 0.71 |
| Main | | 1.27 (1.05;1.54) | 0.016 | 1.13 (1.02;1.26) | 0.017 | 1.22 (1.06;1.40) | 0.006 | 0.91 |
| Main+BMI | | 1.28 (1.05;1.55) | 0.015 | 1.13 (1.02;1.26) | 0.017 | 1.22 (1.06; 1.40) | 0.006 | 0.91 |
|  | |  |  |  |  |  |  |  |

The basic model (n=239,308) was adjusted for age, sex, first 20 genetic principal components and genotyping array. Main model (n=173,387) covariates include basic model covariates + alcohol consumption (g/day), smoking status and Townsend deprivation index. Main+BMI model (n=173,029) covariates include those of the main model and body mass index (BMI).

**Table S7**. Multivariable logistic regression analyses of liver volume associated genetic variants in predicting metabolic dysfunction associated steatotic liver disease (MASLD) and chronic liver disease (CLD) (n=239,308).

|  |  | **Physical Activity** | | | | | | |
| --- | --- | --- | --- | --- | --- | --- | --- | --- |
|  |  | Low (n=28,253) | | Moderate (n=112,364) | | High (n=98,691) | |  |
| Outcome | | OR (95% CI) | *p*-value | OR (95% CI) | *p*-value | OR (95% CI) | *p*-value | *p*_interaction_ |
|  | |  |  |  |  |  |  |  |
| MASLD (n_cases_=172) | |  |  |  |  |  |  |  |
| Basic | | 1.04 (0.84;1.28) | 0.704 | 1.15 (1.03;1.29) | 0.011 | 1.18 (1.02;1.36) | 0.026 | 0.42 |
| Main | | 1.16 (0.87;1.55) | 0.309 | 1.11 (0.96; 1.27) | 0.158 | 1.16 (0.95;1.43) | 0.150 | 0.92 |
| Main+BMI | | 1.16 (0.87:1.54) | 0.327 | 1.10 (0.96;1.26) | 0.179 | 1.16 (0.95;1.42) | 0.145 | 0.91 |
|  | |  |  |  |  |  |  |  |
| CLD (n_cases_=371) | |  |  |  |  |  |  |  |
| Basic | | 1.10 (0.97;1.24) | 0.140 | 1.02 (0.94;1.10) | 0.703 | 1.04 (0.94;1.15) | 0.445 | 0.62 |
| Main | | 0.96 (0.78;1.17) | 0.662 | 0.99 (0.89;1.10) | 0.806 | 1.05 (0.91;1.21) | 0.507 | 0.39 |
| Main+BMI | | 0.96 (0.78;1.17) | 0.663 | 0.98 (0.88;1.10) | 0.772 | 1.05 (0.91;1.21) | 0.506 | 0.39 |
|  | |  |  |  |  |  |  |  |

The basic model (n=239,308) was adjusted for age, sex, first 20 genetic principal components and genotyping array. Main model (n=173,387) covariates include basic model covariates + alcohol consumption (g/day), smoking status and Townsend deprivation index. Main+BMI model (n=173,029) covariates include those of the main model and body mass index (BMI).

**Table S8**. Sensitivity linear regression with robust standard errors analyses of liver fat content associated genetic variants in relation to metabolic dysfunction associated steatotic liver disease (MASLD) and chronic liver disease (CLD) (n=239,308).

|  |  | **Physical Activity** | | | | | | |
| --- | --- | --- | --- | --- | --- | --- | --- | --- |
|  |  | Low (n=28,253) | | Moderate (n=112,364) | | High (n=98,691) | |  |
| Outcome | | Beta (95% CI)* | *p*-value | Beta (95% CI)* | *p*-value | Beta (95% CI)* | *p*-value | *p*_interaction_ |
|  | |  |  |  |  |  |  |  |
| MASLD (n_cases_=172) | |  |  |  |  |  |  |  |
| Basic | | 0.012 (-0.008;0.031) | 0.254 | 0.007 (-0.004;0.018) | 0.190 | 0.011 (0.002;0.019) | 0.019 | 0.89 |
| Main | | 0.001 (-0.016;0.019) | 0.899 | 0.005 (-0.008;0.017) | 0.448 | 0.010 (0.001;0.019) | 0.030 | 0.33 |
| Main+BMI | | 0.001 (-0.017;0.018) | 0.948 | 0.005 (-0.008;0.017) | 0.461 | 0.010 (0.001;0.019) | 0.033 | 0.32 |
|  | |  |  |  |  |  |  |  |
| CLD (n_cases_=371) | |  |  |  |  |  |  |  |
| Basic | | 0.038 (-0.002;0.077) | 0.064 | 0.029 (0.015;0.043) | <0.001 | 0.017 (0.004;0.030) | 0.011 | 0.20 |
| Main | | 0.033 (-0.002;0.068) | 0.067 | 0.014 (0.001;0.028) | 0.062 | 0.016 (0.004;0.029) | 0.012 | 0.54 |
| Main+BMI | | 0.033 (-0.002;0.068) | 0.068 | 0.014 (0.001;0.028) | 0.063 | 0.016 (0.004;0.029) | 0.012 | 0.54 |
|  | |  |  |  |  |  |  |  |

The liver fat associated genetic risk score (GRS_LF_) used in the sensitivity analysis was constructed excluding rs4665985 and rs58542926 as these variants were in linkage disequilibrium with liver volume associated variants rs1260326 (R^2^=0.34) and rs58489806 (R^2^=0.80), respectively. The basic model (n=239,308) was adjusted for age, sex, first 20 genetic principal components and genotyping array. Main model (n=173,387) covariates include basic model covariates + alcohol consumption (g/day), smoking status and Townsend deprivation index. Main+BMI model (n=173,029) covariates include those of the main model and body mass index (BMI).

*Betas and 95% CI limits were multiplied by 100 and reported as percentage.

**Table S9**. Sensitivity linear regression with robust standard errors analyses of liver volume associated genetic variants in relation to metabolic dysfunction associated steatotic liver disease (MASLD) and chronic liver disease (CLD) (n=239,308).

|  |  | **Physical Activity** | | | | | | |
| --- | --- | --- | --- | --- | --- | --- | --- | --- |
|  |  | Low (n=28,253) | | Moderate (n=112,364) | | High (n=98,691) | |  |
| Outcome | | OR (95% CI)* | *p*-value | OR (95% CI)* | *p*-value | OR (95% CI)* | *p*-value | *p*_interaction_ |
|  | |  |  |  |  |  |  |  |
| MASLD (n_cases_=172) | |  |  |  |  |  |  |  |
| Basic | | 0.001 (-0.017;0.019) | 0.956 | 0.009 (-0.001;0.020) | 0.080 | 0.008 (-0.002;0.018) | 0.125 | 0.70 |
| Main | | 0.010 (-0.012;0.031) | 0.381 | 0.005 (-0.006;0.016) | 0.372 | 0.003 (-0.005;0.010) | 0.494 | 0.51 |
| Main+BMI | | 0.009 (-0.012;0.030) | 0.415 | 0.005 (-0.007;0.016) | 0.420 | 0.002 (-0.005;0.010) | 0.536 | 0.53 |
|  | |  |  |  |  |  |  |  |
| CLD (n_cases_=371) | |  |  |  |  |  |  |  |
| Basic | | 0.007 (-0.025;0.039) | 0.650 | 0.004 (-0.011;0.019) | 0.598 | 0.002 (-0.010;0.013) | 0.770 | 0.77 |
| Main | | -0.012 (-0.044;0.019) | 0.442 | -0.002 (-0.018; 0.014) | 0.807 | 0.001 (-0.009;0.010) | 0.879 | 0.39 |
| Main+BMI | | -0.013 (-0.045;0.019) | 0.428 | -0.002 (-0.019;0.014) | 0.767 | 0.001 (-0.009;0.010) | 0.885 | 0.39 |
|  | |  |  |  |  |  |  |  |

The liver volume associated genetic risk score (GRS_LV_) used in the sensitivity analysis was constructed excluding rs1260326 and rs58489806 as these variants were in linkage disequilibrium with liver volume associated variants rs4665985 (R^2^=0.34) and rs58542926 (R^2^=0.80), respectively. The basic model (n=239,308) was adjusted for age, sex, first 20 genetic principal components and genotyping array. Main model (n=173,387) covariates include basic model covariates + alcohol consumption (g/day), smoking status and Townsend deprivation index. Main+BMI model (n=173,029) covariates include those of the main model and body mass index (BMI).

*Betas and 95% CI limits were multiplied by 100 and reported as percentage.

**Table S10**. Sensitivity multivariable logistic regression analyses of liver fat content associated genetic variants in predicting metabolic dysfunction associated steatotic liver disease (MASLD) and chronic liver disease (CLD) (n=239,308).

|  |  | **Physical Activity** | | | | | | |
| --- | --- | --- | --- | --- | --- | --- | --- | --- |
|  |  | Low (n=28,253) | | Moderate (n=112,364) | | High (n=98,691) | |  |
| Outcome | | OR (95% CI) | *p*-value | OR (95% CI) | *p*-value | OR (95% CI) | *p*-value | *p*_interaction_ |
|  | |  |  |  |  |  |  |  |
| MASLD (n_cases_=172) | |  |  |  |  |  |  |  |
| Basic | | 1.13 (0.91;1.41) | 0.269 | 1.09 (0.97;1.23) | 0.147 | 1.22 (1.04;1.42) | 0.012 | 0.48 |
| Main | | 1.01 (0.74;1.38) | 0.936 | 1.07 (0.92;1.24) | 0.361 | 1.33 (1.06;1.67) | 0.012 | 0.15 |
| Main+BMI | | 1.00 (0.73:1.37) | 0.996 | 1.06 (0.92;1.23) | 0.407 | 1.34 (1.07;1.68) | 0.010 | 0.15 |
|  | |  |  |  |  |  |  |  |
| CLD (n_cases_=371) | |  |  |  |  |  |  |  |
| Basic | | 1.16 (1.01;1.32) | 0.030 | 1.20 (1.10;1.30) | <0.001 | 1.16 (1.04;1.29) | 0.006 | 0.94 |
| Main | | 1.25 (1.01;1.55) | 0.038 | 1.12 (1.00;1.25) | 0.055 | 1.23 (1.06;1.44) | 0.007 | 0.90 |
| Main+BMI | | 1.25 (1.01;1.55) | 0.038 | 1.11 (1.00;1.25) | 0.058 | 1.23 (1.06;1.44) | 0.007 | 0.89 |
|  | |  |  |  |  |  |  |  |

The liver fat associated genetic risk score (GRS_LF_) used in the sensitivity analysis was constructed excluding rs4665985 and rs58542926 as these variants were in linkage disequilibrium with liver volume associated variants rs1260326 (R^2^=0.34) and rs58489806 (R^2^=0.80), respectively. The basic model (n=239,308) was adjusted for age, sex, first 20 genetic principal components and genotyping array. Main model (n=173,387) covariates include basic model covariates + alcohol consumption (g/day), smoking status and Townsend deprivation index. Main+BMI model (n=173,029) covariates include those of the main model and body mass index (BMI).

**Table S11**. Sensitivity multivariable logistic regression analyses of liver volume associated genetic variants in predicting metabolic dysfunction associated steatotic liver disease (MASLD) and chronic liver disease (CLD) (n=239,308).

|  |  | **Physical Activity** | | | | | | |
| --- | --- | --- | --- | --- | --- | --- | --- | --- |
|  |  | Low (n=28,253) | | Moderate (n=112,364) | | High (n=98,691) | |  |
| Outcome | | OR (95% CI) | *p*-value | OR (95% CI) | *p*-value | OR (95% CI) | *p*-value | *p*_interaction_ |
|  | |  |  |  |  |  |  |  |
| MASLD (n_cases_=172) | |  |  |  |  |  |  |  |
| Basic | | 1.01 (0.80;1.27) | 0.957 | 1.12 (0.99;1.27) | 0.071 | 1.15 (0.98;1.35) | 0.088 | 0.43 |
| Main | | 1.15 (0.83;1.59) | 0.400 | 1.07 (0.92;1.25) | 0.372 | 1.08 (0.86;1.36) | 0.528 | 0.81 |
| Main+BMI | | 1.13 (0.82;1.56) | 0.461 | 1.06 (0.91;1.24) | 0.439 | 1.08 (0.86;1.35) | 0.527 | 0.86 |
|  | |  |  |  |  |  |  |  |
| CLD (n_cases_=371) | |  |  |  |  |  |  |  |
| Basic | | 1.03 (0.90;1.18) | 0.691 | 1.02 (0.94;1.12) | 0.592 | 1.01 (0.91;1.13) | 0.800 | 0.90 |
| Main | | 0.92 (0.74;1.16) | 0.487 | 0.98 (0.87;1.10) | 0.735 | 1.01 (0.86;1.18) | 0.919 | 0.52 |
| Main+BMI | | 0.92 (0.74;1.15) | 0.473 | 0.98 (0.87;1.10) | 0.680 | 1.01 (0.86;1.18) | 0.924 | 0.51 |
|  | |  |  |  |  |  |  |  |

The liver volume associated genetic risk score (GRS_LV_) used in the sensitivity analysis was constructed excluding rs1260326 and rs58489806 as these variants were in linkage disequilibrium with liver volume associated variants rs4665985 (R^2^=0.34) and rs58542926 (R^2^=0.80), respectively. The basic model (n=239,308) was adjusted for age, sex, first 20 genetic principal components and genotyping array. Main model (n=173,387) covariates include basic model covariates + alcohol consumption (g/day), smoking status and Townsend deprivation index. Main+BMI model (n=173,029) covariates include those of the main model and body mass index (BMI).


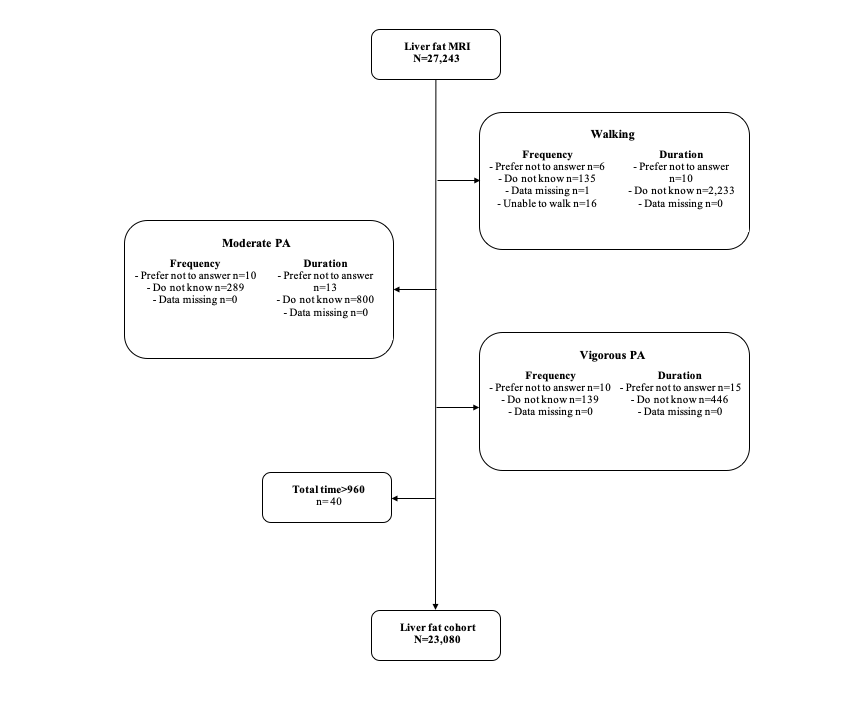


**Figure S1**. Exclusions made in the liver fat cohort based on answers to the International Physical Activity Questionnaire. N: number of individuals in the mentioned cohort, n: number of excluded individuals.


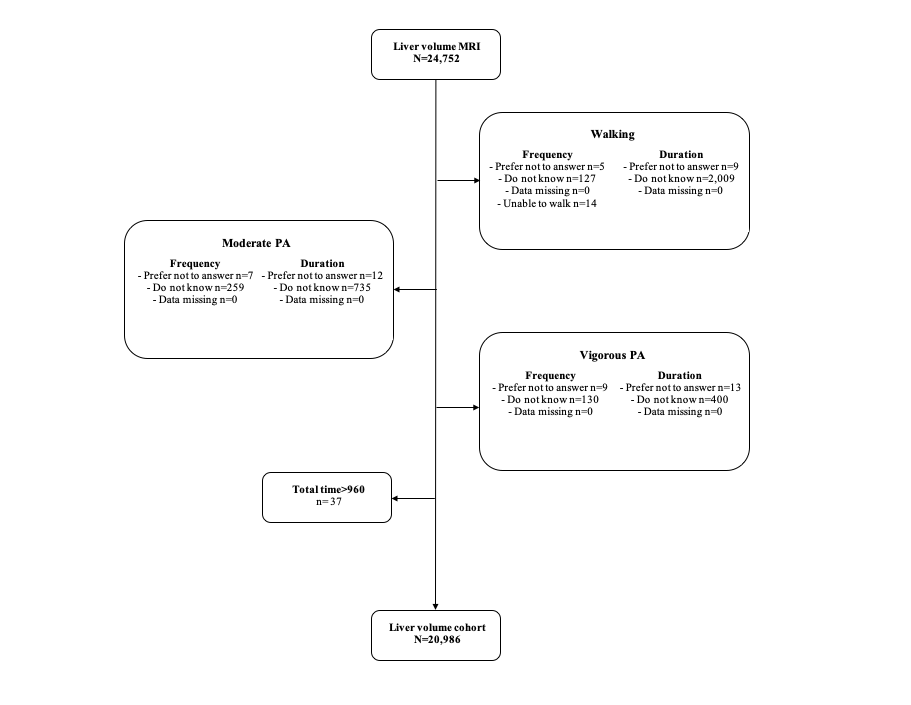


**Figure S2**. Exclusions made in the liver volume cohort based on answers to the International Physical Activity Questionnaire. N: number of individuals in the mentioned cohort, n: number of excluded individuals.
